# The Seven Books of Paulus Ægineta. Translated from the Greek; with a Commentary, Embracing a Complete View of the Knowledge Possessed by the Greeks, Romans, and Arabians

**Published:** 1845-01

**Authors:** 


The Seven Books of Paulus Ægineta. Translated from the Greek; with a Commentary embracing a complete View of the Knowledge possessed by the Greeks, Romans, and Arabians on all Subjects connected with Medicine and Surgery. By *Francis Adams*. In Three Volumes. Vol. 1, pp. 683. Sydenham Society, 1844.

To those who wish to ascertain what the Ancients knew of medicine and surgery; but who have neither time nor power to wade through the numerous and ponderous tomes of antiquity, these volumes will prove a most welcome—perhaps valuable, treasure. We say *perhaps*—for, having explored a considerable number of the ancient writers, from Hippocrates downwards, many years ago, we have formed opinions of our own on the utility of that branch of study. Mr. Adams quotes a curious passage from Rhases, the Arabian, which we shall here notice. "The number of authors is not small by whose labours the art has attained its present growth; and yet one may hope to master the monuments of their industry within the space of a few years. Let us suppose that, in the course of a thousand years, a thousand authors had made improvements in the profession; and then a person who has diligently studied their works may improve his mind as much in knowledge as if he had devoted a thousand years to the study of medicine." *Pref.* To the truth of this passage we entirely object. It is not only untrue, but it is eminently absurd. Is not the study of the old medical writings the study of medicine? He must therefore have meant the thousand years' study of medicine to include the *practice* also. But we fearlessly maintain that the great mass of medical practitioners in this and in every other country, would lose a vast deal of valuable time in the perusal of the ancient medical authors—and that the said time would be far more profitably spent in the acquisition of medical knowledge at the bed-side of sickness, and in attendance on modern lectures. The life of man is three-score years and ten. Out of these, nearly twenty years are devoted to elementary instruction—five or six to professional studies, and the remainder to practice. To attempt a perusal of the Ancients during the five or six years of medical study, would be suicide—and in the intervals of practice, the physician-surgeon, or general practitioner, can rarely do more than keep his information up to the level of advancing science. The pure physician, indeed, while waiting on practice, or rather on Providence, may study the fathers of physick, by whiling away the time that hangs heavily on his hands, in learned leisure; though we are thoroughly convinced that such leisure might be better employed in modern investigation. The fact is that nine-tenths of ancient wisdom, as transmitted by the Fathers, are downright errors—while the one-tenth of truth may be found at home in any good elementary work of our own times.

We are aware that we shall incur the displeasure of the " LAUDATORES TEMPORIS ACTI," and be considered as Goths and Vandals—or Utilitarians; but we care little for these censures. Our observations apply to the study of the medical fathers in their original language and full extent. The abridgement or analysis before us, which will not require more than a few weeks for perusal, has our entire approbation, and is strongly recommended. It is purged of mountains of chaff, though still containing a great deal more than solid grain. It will convey a good idea of what our forefathers of Asia, Greece, Italy, Egypt, &c. knew—and what they did *not* know. The *latter* will strike the modern practitioner much more forcibly than the *former*. It is proper to state that the work of Paulus Ægineta is made the text, or rather the peg on which the author has hung the doctrines and practices of the ancient fathers generally—a plan which required prodigious labour on his own part, and saves a world of toil and time on the part of his readers. PAULUS himself, indeed, was only a COMPILER, according to his own confession, having " set down little of his own:" but he was, on that very account, the very best author that Mr. Adams could have selected for his purpose. We shall now exhibit a few specimens of the wisdom of the Ancients.

1. *Complaints of Pregnancy.*—Redundance of crudities — *continued* vomiting, salivation, heart-burn, &c. *Remedies*—foot exercise—yellow wines that are fragrant, and five years old. *Medicines*—knot-grass—dill —Pontic-root, &c.

2. *Siriasis.*—Inflammation about the brain and its membranes, attended with hollowness of the open of the head and eyes—paleness and dryness of the body. " It is relieved by an application of the red of an egg with oil of roses to the open of the head, in form of compress." p. 16.

3. *Dimness of Sight.*—" In order to avoid dimness of sight, when they plunge into cold water, people ought to open their eyes wide, for thereby the strength of their eyes will be much improved. They ought also to be careful not to hurt them by reading. Let them also avoid wine that is thick and sweet, such articles of food as ascend upwards, whatever is of difficult digestion, and engenders crude and thick humors, the herb rocket, leeks, and everything whose pungency ascends upwards. Let them also avoid reclining long in a supine position, cold, winds blowing direct in the face, smoke and dust; and pour daily into the eyes an infusion prepared thus: for a month and a day, put green fennels into an earthen vessel smeared with pitch on the outside, and pour in rain water, and then taking out the fennels, keep the water laid up for use." 41.

4. *Digestion.*—" Actuarius says, 'I am of opinion, that there are four species of concoction which are performed in different parts of the body: the first in the stomach; the second in the vena ramalis (vena portæ?), meseraic veins, and concave part of the liver; the third in the convex part of the liver and veins proceeding from it; and the fourth, consisting of fabrication or assimilation, which takes place in the extreme parts of the body.' " 91.

5. *Food.*—The ancients, like the moderns, attached considerable importance to diet. They had three or four meals daily. First, the breakfast, *jentaculum*—second, *prandium*, or dinner, about three o'clock—third, *merenda*, the nature of which is not well defined—fourth, *cœna*, or supper, about eight o'clock—and lastly, COMMISSATIO, a kind of jollification after the cœna or great meal. " According to Athenæus, a good physician ought to be a good cook. (Deipnos. vii.) Upon the authority of Daphnus, the Ephesian physician, he decides that night is the most proper time for taking the supper or principal meal, because, says he, the moon promotes putrescency, and digestion is a species of putrefaction." 119.

6. *Flesh.*—Among quadrupeds, swine's flesh is more nourishing than any other food, " because it is most nearly allied to the human, in taste and smell; but the nourishment derived from it is viscid and imperspirable." That from sheep is excrementitious, and " *supplies bad juices.*"

"That from goats is acrid, and has bad juices. But the worst of all is the flesh of the buck-goat as to the quality of its juices and to digestion. That of oxen forms melancholic humors; that of hares has thick juices, but less so than that of sheep and oxen. That of roes is hard and of difficult digestion. In general, the flesh of young beasts is more humid, more tender, and more digestible than that of the aged; of gelded animals than of those having testicles; and of the well-fed than of the lean." 145.

It would be difficult to compress more error into so small a space than the above passage conveys.

7. *Milk.*—When digested, is nutritive, but is injurious to the gums and teeth. It also produces head-ache, flatulence, hypochondria, and engenders stone in the kidneys.

8. *Wine.*—The ancients varied much in their opinions respecting this celebrated beverage. Its moderate use, however, they almost all recommend, " as resuscitating the natural heat within us, improving digestion, and forming good blood." Immoderate drinking is condemned. The following passage is curious.

" When one has drunk largely, it is not proper to take much of any other food; but while drinking, one should eat boiled cabbage, and taste some sweetmeat, particularly almonds. These things relieve headach, and are not difficult to vomit. It is also very proper to take the infusion of wormwood before drinking, for of all things it is the best preservative from surfeit. If one experience any painful effects from wine, one should drink cold water, and the next day again the infusion of wormwood: and by using exercise, friction, the bath, and restricted food, in this way get restored to health." 172.

9. *Fever.*—This disease was considered by the ancients as a preternatural increase of animal heat, diffused from the heart to all parts of the body, through the medium of the arteries. The first thing to be considered is the fatality or non-fatality of the fever—next, whether it will be acute or chronic—and thirdly, whether it will come or not to a crisis. The fatal symptoms are " a death-like countenance—sharp nose—hollow eyes, and other symptoms described by Hippocrates—intolerance of light—one eye less than the other—white of eye appearing red or black—grinding of teeth—delirium—floccitatio, &c." Undoubtedly it requires no ghost or prophet to tell that these are bad symptoms when the fever has progressed to this state; but how is the prognosis to be formed at the *beginning* of the fever? As for the other two questions, they are answered in such a vague or erroneous manner as to require no comment.

10. *The Pulse.*—" The pulse is a movement of the heart and arteries, taking place by a diastole and systole. Its object is twofold; for, by the diastole, which is, as it were, an unfolding and expansion of the artery, the cold air enters, ventilating and resuscitating the animal vigour, and hence the formation of the vital spirits; and by the systole, which is, as it were, a falling down and contraction of the circumference of the artery towards the centre, the evacuation of the fuliginous superfluities is effected." 202.

11. *Cure of Tertian Fever.*—" In the true tertian fever, as arising from yellow bile, we must dilute and cool—evacuate the defluxions upon the stomach by emetics—and downwards by the bowels, carrying them off by perspiration and urine." When symptoms of concoction appear, wormwood may be given.

12. *Love-Sickness.*—" It will not be out of place here to join love to the affections of the brain, since it consists of certain cares. For care is a passion of the soul occasioned by the reason's being in a state of laborious emotion. The following symptoms attend lovers: Their eyes are hollow, and do not shed tears, but appear as if overflowing with gladness, their eyelids move rapidly; and even, when none of the other parts of the body are affected, these parts are always so affected in lovers. There is no pulse peculiar to lovers, as some have supposed, but it is the same as that of persons labouring under care. When they call to recollection the beloved object, either from seeing or hearing, and more especially if this occur suddenly, then the pulse undergoes a change from the disorder of the soul, and, therefore, it does not preserve its natural equability or order. Such persons, therefore, being desponding and sleepless, some physicians, mistaking their affection, have wasted them by prohibiting baths, and enjoining quietude, and a spare diet; but wiser ones, recognizing the lover, direct his attention to baths, the drinking of wine, gestation, spectacles, and amusing stories."

13. *Spleen.*—" The use of the spleen being to attract from the liver the melancholic humour, which is, as it were, the lees of the blood, if its attractive power be weakened, or the passage obstructed by which this was formerly attracted, the black jaundice is formed, blood in an unpurified state being distributed over the whole body; and if there be weight and distension about the spleen, or if there be also pain, obstruction is indicated; but if there be none of these, it is weakness of the attractive power. But vomiting of black bile taking place without fever, or any other symptom of malignity, indicates a weakness of the retentive faculty of the spleen. A weakness of the expulsive faculty will bring on anorexia, the melancholic superfluity being no longer carried to the orifice of the stomach and exciting the appetite." 577.

Reader! have you enough of these specimens of ancient medical knowledge ? If so, shut the book, and if a student, go to the hospital, the lecture-room, and the dissecting-room for information, rather than to Galen and Paulus Ægineta. If you are in actual practice, study diseases for yourself at the bedside of sickness, and keep up to the level of the swelling tide of knowledge that everywhere flows around you. If, however, you prefer the imperfect—too often absurd, doctrines and practices of the Arabian, Greek, and Roman physicians, you will study their writings by day and by night, till you have earned for yourself the reputation of being a learned veteran of—THE OLD SCHOOL.

